# Effects of Chinese pickled and dried mustard on nutritional quality, sensory quality, and shelf life of steamed pork belly

**DOI:** 10.1002/fsn3.612

**Published:** 2018-03-08

**Authors:** Qing Shen, Mengting Wang, Jinhu Tian, Lyulin Hu, Sijie Ren, Jianchu Chen, Xingqian Ye, Donghong Liu

**Affiliations:** ^1^ Department of Food Science and Nutrition Zhejiang Key Laboratory for Agro‐Food Processing College of Biosystems Engineering and Food Science Fuli Institute of Food Science Zhejiang R&D Center for Food Technology and Equipment Zhejiang University Hangzhou China

**Keywords:** fatty acid, lipid oxidation, pickled and dried mustard, protein oxidation, shelf life

## Abstract

Steamed pork with pickled and dried mustard (PDM) is a famous Chinese dish. Here, we examined the effects of PDM on nutritional quality, sensory quality, and shelf life of steamed pork belly. Proximate composition, lipid oxidation, fatty acid profiles, protein hydrolysis and oxidation, sensory evaluation, and induction period (IP) of steamed pork belly were determined after addition of different levels (0–100%, *W*_PDM_/*W*_pork belly_) of PDM. The results demonstrated that PDM could significantly (*p *<* *.05) enhance the loss of moisture and fat, increase the ratio of unsaturated to saturated fatty acids, and decrease lipid and protein oxidation in steamed pork belly. Additionally, IP values and steamed pork belly preservation times increased as the amount of added PDM increased. Best sensory quality was achieved when moderate levels of PDM (40%) were added to steamed pork belly. These findings provide insights into the beneficial effects of PDM on steamed pork belly.

## 1 INTRODUCTION

Pickled and dried mustard (PDM; also known as Meigan cai in China) is a fermented vegetable product that is widely consumed in southern China, particularly in the Yangtze River Delta Megalopolis (Li et al., 2012). PDM is traditionally homemade and produced as described by Huang, Huang, and Feng (2012). As a food ingredient with aromatic flavor, PDM is widely added to pork, chicken, fish, and cowpeas during cooking by various methods, including steaming and stir‐frying. In particular, PDM with minced pork is often used for making dumplings, steamed buns, and pancakes, which are very popular in China.

Chinese food culture is well known throughout the world, and steamed pork with PDM is a famous dish that is delicious. Steamed pork with PDM is prepared using pork belly and PDM with little additives and is cooked by steaming. This food product has great taste and flavor and provides a sense of fullness after consumption. Moreover, Xu (1996) reported that steamed pork with PDM had a much longer shelf life than that without PDM and could be stored for more than half a year at room temperature. These properties are thought to be related to the potential antioxidant activity of PDM; indeed, Li et al. (2012) confirmed that PDM extract contains gallic and protocatechuic acids, which could be used as a potential source of natural antioxidants. Wang (2013) investigated the antimicrobial and antioxidant properties of PDM and its application in pork preservation. The results showed that PDM extract promoted fresh pork preservation. However, to the best of our knowledge, few reports have focused on the effects of PDM on steamed pork. The changes in chemical components in steamed pork with PDM, which are closely related to its nutritive, organoleptic, and preservative properties, are still unclear.

Fat and protein are the main nutritional components in pork belly, and their oxidative and hydrolytic reactions in meat are the most important factors affecting quality and shelf life. Ali et al. (2015) reported that deterioration of frozen meat during storage occurs because of lipid and protein degradation. Oxidative reactions occur during storage and processing of meat and meat products, and processing steps such as mincing, cooking, and salt addition, which promote the formation of reactive oxygen species (ROS) and increase the susceptibility of products to oxidation (Nakyinsige et al., 2015). Antioxidants such as gallic and protocatechuic acids present in PDM have the ability to absorb ROS and suppress or inhibit peroxidation and oxidation of lipids and proteins (Harlina et al., 2015). There is a fine relationship between the degree of oxidation in meat and its shelf life. Therefore, it is necessary to evaluate lipid and protein oxidation, including fatty acid profiles of steamed pork belly, to determine the effects of PDM on meat.

Accordingly, in this study, we aimed to evaluate the effects of Chinese PDM on nutritional quality, sensory quality, and shelf life of steamed pork belly, which may provide useful information for preparation of the Chinese traditional steamed pork with PDM, both for domestic cooking and industrialized production. Additionally, the findings of this study may provide a foundation for expanding the applications of PDM for economic benefits.

## 2 MATERIALS AND METHODS

### 2.1 Raw materials and sample preparation

The brand of Xian Heng PDM (Meigan cai) was obtained from Xianheng Foodstuffs Co., Ltd (Shaoxing, China). Pork belly (lean/fat ratio of about 1:2) was purchased from Wal‐Mart Supermarket in Hangzhou, Zhejiang Province, China.

Steamed pork with PDM was prepared according to Chinese traditional domestic cooking methods as described by Liu, Jiang, Lian, Zhang, and Ma (1999), with some modifications. Pork belly (about 250 g per replicate in a bowl) was randomly selected and precooked in boiling water (pork:water ratio of 1:4, w/v) for 1 min and then cut into 3 × 4 × 1‐cm^3^ pieces. Five grams of sugar was added with 0, 50, 100, 150, 200, or 250 g PDM to each bowl, and all materials were then mixed evenly. The entire bowl was then placed in a pressure cooker (Supor, China) for 20 min for high‐pressure steaming on an induction cooker (2,300 W), followed by cooling for 5 min. The pressure cooker was then opened, and the pork belly was separated from each bowl. The pork belly was used for texture profile analysis and sensory evaluation after cooling to room temperature or was minced with a mincing machine (Joyoung, China) and then stored at −20°C for further determination of other physical and chemical properties.

Pork belly samples were divided into seven groups as follows: the control group, comprising raw pork belly; steamed pork belly alone; steamed pork belly treated with 20% (50 g) PDM; steamed pork belly treated with 40% (100 g) PDM; steamed pork belly treated with 60% (150 g) PDM; steamed pork belly treated with 80% (200 g) PDM; and steamed pork belly treated with 100% (250 g) PDM. All treatments for each determination were carried out in triplicate.

### 2.2 Analysis of proximate composition

Moisture content was determined using a halogen moisture detector (DHS‐20A; Shanghai Eastsen Analytical Instrument Co., Ltd, China). Crude protein, ash, and sodium chloride contents were determined according to the National Standard of China. Briefly, the crude protein content was determined using the Kjeldahl procedure with a nitrogen‐to‐protein conversion factor of 6.25 (GB5009.5‐2010). The ash content was determined by heating 3 g samples in a furnace at 550°C for about 4 hr until a constant weight was obtained (GB5009.4‐2010). Sodium chloride content was determined according to the silver nitrate titration method (GB/T 12457‐2008).

### 2.3 Total lipid extraction and content analysis

Total lipids were extracted following the method of Folch, Lees, and Stanley (1957), with some modifications. Briefly, the minced pork belly was homogenized with a 2:1 chloroform–methanol mixture to a final dilution of 10‐fold the volume of the sample and extracted in a water bath for 15 min at 60°C, followed by filtration. The extracted lipids were purified by evaporating the solution under vacuum conditions, using a rotary evaporator (RE‐52AA; Yarong Biochemical Analysis Co., Ltd, China), and then stored at −80°C for further analysis or dried at 100°C for about 2 hr to a constant weight to determine the total lipid content, expressed as g/100 g fresh sample. Additionally, chloroform was replaced with dichloromethane in the experiment to reduce toxicity.

### 2.4 Fatty acid analysis

The fatty acid composition of pork belly was determined by gas chromatography (GC). The methyl ester preparation method was carried out as described by Ichihara, Shibahara, Yamamoto, and Nakayama (1996) and Zhang, Wu, et al. (2013). Briefly, 10 mg of extracted lipids was dissolved in 2 ml hexane and then added to 4 ml of 2 mol/L methanolic KOH. The mixture was then shaken for 30 min at 25°C, and the hexane layer was used for GC analysis, which was performed using a GC7890A gas chromatograph (Agilent Technologies Inc.) equipped with an automatic sampler, a split/splitless injector, a fused silica capillary column (DB‐23; 30 m long × 0.32 mm id × 0.25 μm film thickness; Agilent Technologies Inc.), and a flame ionization detector. Nitrogen was used as the carrier gas with the flow rate set at 9 ml/min, the pressure at 6.6016 psi, and a split ratio of 5:1. The temperature of the flame ionization detector was maintained at 280°C, and that of the injector was maintained at 270°C. The column temperature was programmed as follows: maintained at 120°C for 5 min, increased from 120 to 190°C at 5°C/min, maintained at 190°C for 12 min, increased to 210°C at 2.5°C/min, and finally maintained at 210°C for 10 min. A 37 fatty acid methyl ester (FAME) mixture standard (Sigma‐Aldrich Co. LLC.) was run under the same conditions.

### 2.5 Lipid oxidation measurement

Thiobarbituric acid (TBA) and peroxide value (POV) were determined to evaluate the extent of lipid oxidation. TBA measurement was performed as described by Sorensen and Jorgensen (1996), with some modifications. Briefly, the minced sample (10 g) was homogenized (15,000 rpm, 30 s) with 50 ml of a 7.5% trichloroacetic acid (TCA) solution containing 0.1% ethylenediaminetetraacetic acid, using a homogenizer (FSH‐2A; Jiangsu Zhenxing Instrument Factory, China). After filtration, 5.0 ml extract was mixed with 5.0 ml of 0.02 mol/L aqueous TBA in stoppered glass tubes and incubated in a water bath at 100°C for 1 hr and then cooled for 10 min in cold water. Absorbance was measured at 532 nm using a UV spectrophotometer (2550; Shimadzu, Japan). The results were calculated from the standard curve of 1,1,3,3‐tetraethoxypropane and expressed as milligrams of malondialdehyde (MDA) per kilogram of pork belly sample. POV was assessed according to AOAC method 965.33 and expressed as milliequivalents (meq) of peroxide per kilogram of pork belly sample.

### 2.6 Protein hydrolysis and oxidation measurement

#### 2.6.1 Soluble protein extraction and concentration determination

The soluble protein of pork belly was extracted with sodium phosphate buffer. Briefly, 2 g minced pork belly was homogenized (15,000 rpm, 30 s) with 10 ml of 20 mmol/L sodium phosphate buffer (pH 6.8), extracted for 1 hr at 4°C, and then filtered with Whatman No. 1 filter paper. The filtrate was preserved at −20°C for further determination. The protein concentration of the filtrate was determined using a BCA kit (Sigma), and bovine serum albumin was used as the standard.

#### 2.6.2 Protein hydrolysis measurement

The degree of hydrolysis of the protein sample was determined by the trinitrobenzenesulfonic acid (TNBS) method, as described by Adlernissen (1979), with minor modifications. Briefly, 0.020 ml of the protein extraction sample was mixed in a test tube with 0.980 ml sodium phosphate buffer (pH 8.0). One milliliter of 1.0% TNBS solution was added, and the test tubes were shaken and placed in a water bath at 50°C for 60 min. After 60 min, 2.0 ml of 0.1 N HCl was added to terminate the reaction, and the absorbance was then read against water at 340 nm. The degree of hydrolysis of the protein sample was expressed as millimoles of free amino groups per gram of pork belly soluble protein, and glycine was used to construct a standard curve.

#### 2.6.3 Protein oxidation measurement

Protein carbonyl and sulfhydryl measurements are common methods for determining protein oxidation. The protein carbonyls were determined by reacting proteins with 2,4‐dinitrophenylhydrazine (DNPH), as described by Ali et al. (2015), with minor modifications. Briefly, a 0.7‐ml protein sample extract was reacted with 0.3 ml of 10 mmol/L DNPH for 1 hr at room temperature. Proteins were precipitated with 1 ml of 40% TCA and washed three times with 1.0 ml of an ethanol/ethyl acetate mixture (1:1). The protein was dried and dissolved in 3 ml of 6 mol/L guanidine hydrochloride. The absorbance was measured at 370 and 280 nm to determine carbonyl and protein contents, respectively. Results are expressed as the ratio of the absorbance at 370 nm to that at 280 nm. The sulfhydryl content was determined by reacting with 5,5′‐dithiobis(2‐nitrobenzoic acid) (DTNB) according to Srinivasan and Hultin (1997), with some modifications. One milliliter of the protein sample extract was transferred to a tube and dissolved in 2 ml sodium phosphate buffer (pH 8.0). The mixture was then incubated with 0.5 ml of 10 mmol/L DTNB reagent at room temperature away from light for 1 hr, and the absorbance was measured against phosphate buffer at 412 nm. The results were expressed as millimoles of total free sulfhydryl groups per gram of pork belly soluble protein.

### 2.7 Texture profile analysis (TPA)

Texture profile analysis was carried out as described by Bourne (1978). A texture analyzer (TA‐XT2i 2; Stable Micro Systems, Surrey, UK) with a 5‐mm‐diameter cylindrical probe was used. The samples were compressed to 50% of their original height in a two‐cycle compression using a crosshead speed of 1.0 mm/s. The cubic muscle layer and fat layer (1 × 1 × 1‐cm^3^) cut from pork belly samples were determined five times for every treatment. Hardness, springiness, cohesiveness, gumminess, chewiness, and resilience parameters were calculated for each sample.

### 2.8 Sensory evaluation

Quantitative descriptive analysis for sensory evaluation was carried out as described by Morita, Kubota, and Aishima (2003), with some modifications. Briefly, 16 panelists (eight men and eight women, ages 20–30 years) with previous experience in food sensory evaluation participated in the study. Each member was randomly given one 10‐g pork belly sample from each group. Samples were spread on glass plates, and a glass of water was provided for gargling between samples. Panelists evaluated the samples for four different attributes (color, aroma, flavor, and texture) and graded each sample (on a scale of 1–25) for each attribute according to the standard description of steamed pork with PDM. Higher points indicated better sensory qualities closer to the standard description. Evaluations occurred in individual rooms under white light at a temperature of 22–25°C.

### 2.9 OXITEST analysis

Oxidation tests were performed using an OXITEST reactor (Velp Scientifica, Usmate, Milan, Italy), as described by Verardo et al. (2013), with some modifications. Six grams of minced pork belly was used for analysis in each chamber. The conditions were set at 80, 90, and 100°C, with an initial oxygen pressure of 6 bar. Each sample was measured four times for each treatment, and the induction periods (IPs) were calculated using the OXITEST reactor.

### 2.10 Statistical analysis

All experiments were carried out in triplicate (*n *= 3). Results were expressed as means ± standard deviations. Treatment effects were analyzed by one‐way analysis of variance (ANOVA), using SPSS 20.0 (SPSS Inc., Chicago, IL). Duncan's multiple range tests were performed to determine mean separation. Differences with *p* values of less than .05 were considered statistically significant and those of less than .01 were considered statistically highly significant.

## 3 RESULTS AND DISCUSSION

### 3.1 Proximate composition

The results for proximate composition of raw and steamed pork belly in the presence of PDM are shown in Table 1. The moisture, crude protein, total lipid, ash, and sodium chloride contents of the raw pork belly sample were 36.92, 20.47, 39.30, 1.10, and 0.20 g/100 g fresh weight, respectively. After steaming, the moisture and crude protein contents of pork belly increased significantly (*p < *.05), the total lipids decreased significantly (*p *<* *.05) to 33.24%, and the ash and sodium chloride contents were not significantly (*p *>* *.05) altered. This trend was consistent with that in a report by Zhang, Wu, et al. (2013), who demonstrated that steamed cooking affected the chemical composition of fish fillets. The increased moisture could be explained by the steaming process, which involves the use of steam from heated water for cooking and may cause samples to hold more moisture. Li, Li, Li, et al. (2016) also observed a decrease in total fat in pork belly samples after cooking and explained this change by the melting and liquefaction of fat during cooking, which may also cause increased moisture and crude protein percentages.

**Table 1:** Effects of Chinese pickled and dried mustard on the proximate composition of steamed pork belly (g/100 g fresh weight)

Treatments	Moisture	Crude protein	Total lipids	Ash	Sodium chloride
Control (raw)	36.92 ± 0.84^c^	20.47 ± 1.26^d^	39.30 ± 1.15^a^	1.10 ± 0.01^e^	0.20 ± 0.01^a^
0% PDM	42.28 ± 1.26^a^	22.95 ± 0.71^c^	33.24 ± 0.32^b^	1.24 ± 0.04^e^	0.20 ± 0.01^a^
20% PDM	39.61 ± 0.42^b^	24.41 ± 0.58^bc^	33.99 ± 1.01^b^	1.77 ± 0.04^d^	0.82 ± 0.05^b^
40% PDM	40.26 ± 1.27^b^	24.47 ± 1.05^bc^	32.74 ± 0.79^bc^	1.93 ± 0.04^c^	0.81 ± 0.07^b^
60% PDM	39.84 ± 0.83^b^	25.66 ± 0.62^b^	31.21 ± 1.47^c^	2.02 ± 0.13^c^	0.76 ± 0.01^b^
80% PDM	37.93 ± 0.36^c^	27.39 ± 1.20^a^	29.58 ± 0.72^d^	2.52 ± 0.09^b^	0.84 ± 0.05^b^
100% PDM	37.85 ± 0.54^c^	28.36 ± 1.20^a^	28.02 ± 0.56^d^	3.00 ± 0.15^a^	0.84 ± 0.05^b^

Different letters within a column are significantly different (*p *<* *.05).

Compared with that in steamed pork belly without PDM, the addition of PDM decreased moisture and total lipid contents. The moisture content of samples after addition of PDM was 37.85–40.26 g/100 g fresh weight and decreased as the amount of PDM increased. When 80% PDM or more was added, the moisture content did not differ significantly (*p *>* *.05) from that of the raw sample, that is, addition of 80% or more PDM prevented pork belly samples from holding moisture during steaming. This result could be explained by the physical barrier function and water‐absorbing capacity (diffusion motivated) of PDM, attributed to the physicochemical characteristics of the dehydrated vegetable. Additionally, PDM also had lipid‐binding activity, decreasing the total lipid contents of pork belly from 33.99 to 28.02 g/100 g fresh weight. Thus, PDM, which covered the pork belly during steaming, served as an absorbent medium to lower moisture and fat contents of pork belly, which could explain the extended shelf life of steamed pork.

As shown in Table 1, crude protein and ash contents tended to increase as the amount of PDM increased from 0% to 100% as a result of decreased moisture and total lipid contents. The sodium chloride content increased significantly (*p *<* *.05) in samples treated with PDM compared with those in untreated samples; however, this difference was not concentration dependent. PDM is a type of homemade preserved vegetable, and a small amount of pure, granulated, noniodized pickling salt is added during preparation (Huang et al., 2012). Thus, some of the salt may have diffused into the pork belly during the steaming process as no extra salt was added in the experiment. Nevertheless, 20% PDM treatment made the pork belly sufficiently salty (0.76–0.84 g/100 g fresh weight, around four times that of the raw or untreated sample), and higher amounts of added PDM did not have additional effects. Because sodium chloride plays an important role in meat preservation (Verma & Banerjee, 2012), steamed pork with PDM treatment could be stored longer than that without PDM treatment.

### 3.2 Lipid oxidation

Peroxide value is the primary product of lipid oxidation generated by attack of oxygen on the double bond in fatty acids, and TBA represents the content of secondary lipid oxidation products, primarily aldehydes, which contribute to off‐flavors in meat (Cao et al., 2013). POV and TBA are commonly used as indicators of lipid oxidation and are extensively used to detect oxidative deterioration of fat‐containing foods (Harlina et al., 2015).

**Figure 1:**
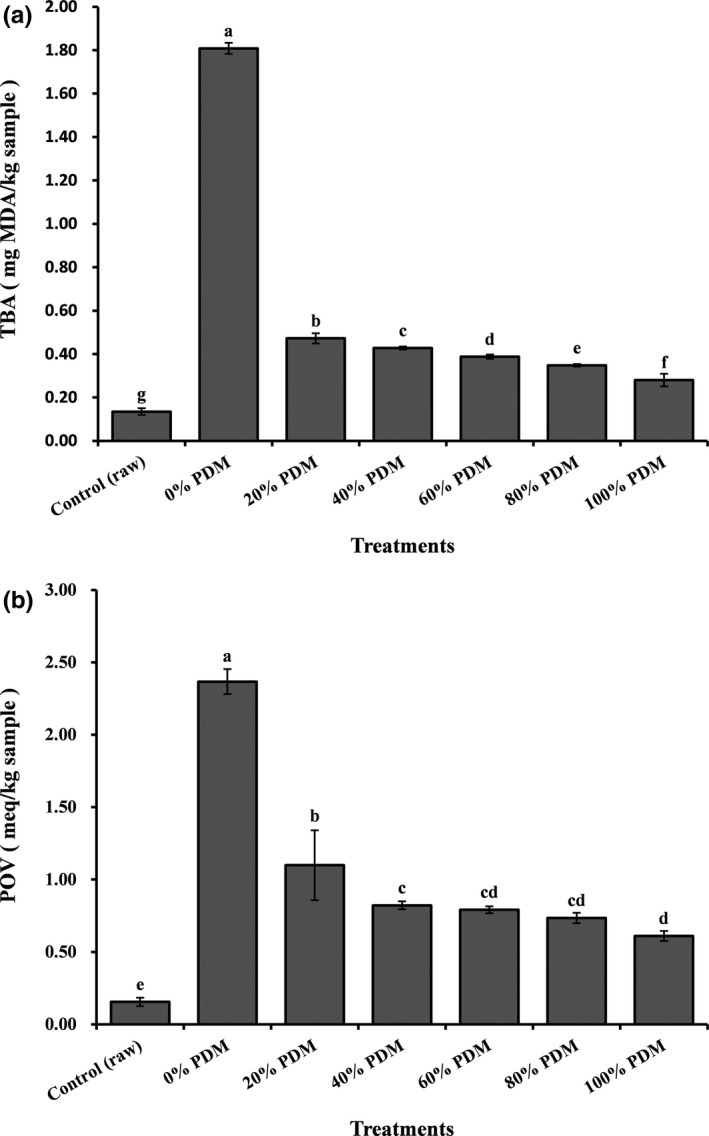
Thiobarbituric acid (TBA) values (a) and peroxide values (POVs, b) of steamed pork belly with different levels of added Chinese pickled and dried mustard (PDM). Values are presented as means ± SDs. Bars with different letters are significantly different (*p *<* *.05)

In this study, the TBA (Figure 1a) and POV (Figure 1b) of steamed pork belly were significantly (*p *<* *.05) affected by the addition of PDM and exhibited similar trends. The POV and TBA of the raw pork belly sample were 0.16 meq/kg sample and 0.14 mg MDA/kg sample, respectively, and increased to 2.37 meq/kg sample and 1.81 mg MDA/kg sample after steaming (no PDM treatment). This was consistent with a report by Bakar, Rahimabadi, and Man (2008), who showed that cooking increased the POV and TBA of samples. Compared with those of untreated but steamed samples, samples treated with added PDM exhibited significantly (*p < *.05) decreased POVs (from 1.10 to 0.61 meq/kg sample) and TBA values (from 0.47 to 0.28 mg MDA/kg sample) as the level of added PDM increased from 20% to 100%, indicating a strong resistance to lipid oxidation in a PDM quantity‐dependent manner. The relatively low extent of lipid oxidation in PDM‐treated samples may have been due to the antioxidative activity of PDM, as reported by Huang et al. (2012) and Li et al. (2012), and the relatively low proportion of fat exposed to oxygen (pork belly was covered with PDM, and increased PDM resulted in lower proportion of fat exposed to oxygen). Therefore, PDM could extend the shelf life of steamed pork belly by reducing the degree of lipid oxidation.

### 3.3 Fatty acid profile

Table 2 shows that the content of saturated fatty acids (SFAs; 39.88%) in the raw pork belly samples was the highest, followed by monounsaturated fatty acids (MUFAs; 36.93%) and polyunsaturated fatty acids (PUFAs; 23.19%). The most abundant fatty acids found in raw samples were palmitic acid (C16:0; 22.84%), stearic acid (C18:0; 15.44%), oleic acid (C18:1n9c; 34.60%), and linoleic acid (C18:2n6c; 18.95%). Our data on SFAs and their composition were consistent with those reported by Guillevic, Kouba, and Mourot (2009), Li, Li, Li, (2016), and Park, Yoo, Shim, and Chin (2008); however, the content of linoleic acid was higher, whereas the content of oleic acid was lower in our study. In comparison with the control group and untreated steamed samples, steaming resulted in a significant decrease in PUFAs and a significant increase in MUFAs and SFAs (*p *<* *.05), which could be explained by the observation that degradation of long‐chain PUFAs may contribute to the increase in medium/short‐chain MUFAs and SFAs during high‐pressure steaming. However, Li, Li, Zhao, et al. (2016) showed that processing resulted in a reduction in total SFAs (*p *<* *.01) in all processing treatments compared with that in raw pork belly samples due to the melting of SFAs into broth after prolonged heating. Moreover, Zhang, Wu, et al. (2013) found that steaming did not significantly (*p *>* *.05) affect the fatty acid composition of grass carp fillets but caused a minor increase in SFAs. Thus, rather than sample characteristics or cooking methods, the detail of the heating process seemed to be important for determining the composition of fatty acids.

**Table 2:** Effects of Chinese pickled and dried mustard on fatty acid profiles of steamed pork belly (area % of total fatty acids)

Retention time (min)	Fatty acid	Treatments						
		Control (raw)	0% PDM	20% PDM	40% PDM	60% PDM	80% PDM	100% PDM
9.44	C12:0	0.06 ± 0.01^a^	0.06 ± 0.06^a^	0.11 ± 0.03^a^	0.06 ± 0.02^a^	0.11 ± 0.03^a^	0.11 ± 0.01^a^	0.07 ± 0.01^a^
13.14	C14:0	0.95 ± 0.01^c^	0.96 ± 0.02^c^	0.95 ± 0.01^c^	0.96 ± 0.00^c^	1.06 ± 0.04^b^	1.15 ± 0.00^a^	1.08 ± 0.01^b^
16.58	C16:0	22.84 ± 0.13^c^	24.16 ± 0.55^a^	23.51 ± 0.07^b^	23.17 ± 0.60^bc^	21.60 ± 0.16^d^	21.55 ± 0.05^d^	21.22 ± 0.12^d^
18.12	C17:0	0.30 ± 0.00^a^	0.28 ± 0.00^ab^	0.28 ± 0.00^ab^	0.26 ± 0.02^b^	0.18 ± 0.03^c^	0.15 ± 0.01^d^	0.15 ± 0.00^d^
19.68	C18:0	15.44 ± 0.06^a^	16.10 ± 0.77^a^	15.59 ± 0.18^a^	15.42 ± 0.43^a^	12.70 ± 0.44^b^	10.92 ± 0.02^c^	11.01 ± 0.14^c^
23.52	C20:0	0.30 ± 0.00^ab^	0.25 ± 0.06^ab^	0.22 ± 0.09^b^	0.27 ± 0.01^ab^	0.35 ± 0.07^a^	0.30 ± 0.04^ab^	0.27 ± 0.01^ab^
	**∑SFA**	**39.88 ± 0.18** ^**b**^	**41.82 ± 1.18** ^**a**^	**40.65 ± 0.19** ^**b**^	**40.15 ± 0.25** ^**b**^	**35.99 ± 0.46** ^**c**^	**34.18 ± 0.00** ^**d**^	**33.80 ± 0.26** ^**d**^
15.57	C15:1	0.09 ± 0.02 ^cd^	0.21 ± 0.08^ab^	0.22 ± 0.05^a^	0.16 ± 0.01^abc^	0.14 ± 0.04^bcd^	0.09 ± 0.03 ^cd^	0.06 ± 0.00^d^
17.02	C16:1	1.26 ± 0.00^f^	1.61 ± 0.04 ^cd^	1.58 ± 0.01^d^	1.67 ± 0.04^b^	1.47 ± 0.03^e^	1.82 ± 0.01^a^	1.63 ± 0.00^bc^
18.58	C17:1	0.16 ± 0.00^ab^	0.16 ± 0.02^ab^	0.13 ± 0.06^ab^	0.18 ± 0.01^a^	0.16 ± 0.03^ab^	0.14 ± 0.02^ab^	0.12 ± 0.00^b^
20.06	C18:1n9c	34.60 ± 0.17^d^	37.21 ± 0.83^c^	37.82 ± 0.08^c^	38.61 ± 0.53^b^	37.86 ± 0.28^c^	39.95 ± 0.08^a^	39.61 ± 0.25^a^
24.10	C20:1	0.83 ± 0.01^b^	0.88 ± 0.02^ab^	0.92 ± 0.00^a^	0.88 ± 0.07^ab^	0.73 ± 0.08^c^	0.70 ± 0.05^c^	0.68 ± 0.01^c^
	**∑MUFA**	**36.93 ± 0.18** ^**d**^	**40.07 ± 0.83** ^**c**^	**40.67 ± 0.02** ^**c**^	**41.50 ± 0.49** ^**b**^	**40.35 ± 0.24** ^**c**^	**42.70 ± 0.02** ^**a**^	**42.10 ± 0.26** ^**ab**^
20.48	C18:2n6t	2.03 ± 0.01^b^	2.43 ± 0.05^a^	2.42 ± 0.01^a^	2.39 ± 0.08^a^	0.09 ± 0.06^c^	0.11 ± 0.02^c^	0.08 ± 0.01^c^
20.90	C18:2n6c	18.95 ± 0.02^d^	13.71 ± 0.28^e^	14.10 ± 0.02^e^	13.97 ± 0.22^e^	20.77 ± 0.49^b^	20.27 ± 0.07^c^	21.20 ± 0.02^a^
22.10	C18:3n3	0.88 ± 0.01^b^	0.74 ± 0.02^c^	0.76 ± 0.00^c^	0.77 ± 0.01^c^	1.47 ± 0.04^a^	1.46 ± 0.02^a^	1.49 ± 0.01^a^
25.46	C20:2	0.89 ± 0.01^ab^	0.68 ± 0.02^d^	0.73 ± 0.01^c^	0.74 ± 0.05^c^	0.92 ± 0.01^a^	0.87 ± 0.01^b^	0.93 ± 0.01^a^
26.85	C20:3n3	0.35 ± 0.01^b^	0.50 ± 0.05^a^	0.52 ± 0.10^a^	0.37 ± 0.02^b^	0.22 ± 0.02^c^	0.23 ± 0.00^c^	0.21 ± 0.00^c^
27.40	C20:4n6	0.09 ± 0.02^ab^	0.04 ± 0.04^b^	0.14 ± 0.17^ab^	0.11 ± 0.02^ab^	0.20 ± 0.00^a^	0.18 ± 0.00^a^	0.20 ± 0.00^a^
	**∑PUFA**	**23.19 ± 0.03** ^**b**^	**18.11 ± 0.36** ^**d**^	**18.68 ± 0.19** ^**c**^	**18.35 ± 0.27 ** ^**cd**^	**23.66 ± 0.46** ^**a**^	**23.12 ± 0.03** ^**b**^	**24.10 ± 0.02** ^**a**^
	**∑UFA**	**60.12 ± 0.18** ^**c**^	**58.18 ± 1.18** ^**d**^	**59.35 ± 0.19** ^**c**^	**59.35 ± 0.19** ^**c**^	**59.85 ± 0.25** ^**b**^	**65.82 ± 0.00** ^**a**^	**66.20 ± 0.26** ^**a**^
	**UFA/SFA**	**1.51 ± 0.01** ^**c**^	**1.39 ± 0.07** ^**d**^	**1.46 ± 0.01** ^**c**^	**1.49 ± 0.02** ^**c**^	**1.78 ± 0.04** ^**b**^	**1.99 ± 0.00** ^**a**^	**2.02 ± 0.02** ^**a**^

Different letters within a row are significantly different (*p *<* *.05).

MUFA, monounsaturated fatty acid; PUFA, polyunsaturated fatty acid; SFA, saturated fatty acid; UFA, unsaturated fatty acid.

PDM treatment affected the composition of fatty acids in our study. MUFAs (40.67%–42.10%) made up the bulk of the fatty acids in PDM‐treated samples rather than SFAs (33.80%–40.65%), and the PUFA content (18.35%–24.10%) was as low as that in the control group and untreated steamed samples. As the amount of PDM increased from 0% to 100%, the evolution of unsaturated fatty acids (UFAs; 58.18%–66.20%) was opposite that of SFAs (41.82%–33.80%); thus, the UFA‐to‐SFA ratio changed from 1.39 to 2.02. Some fatty acids played important roles in this trend, with decreases in C16:0, C17:0, and C18:0 and increases in C18:1n9c, C18:2n6c, C18:3n3, and C20:2. We assumed that increases in UFA contents during heat treatment could be attributed to the high levels of natural antioxidants present in steamed pork with PDM, such as Maillard reaction products (particularly melanoidins), which may inhibit autoxidation of the UFAs during processing (Li, Li, Zhao, et al., 2016). Nonetheless, the contents of MUFAs and PUFAs did not show a gradual increase. When adding 60% PDM, the MUFA content was 40.35%; this value was not significantly (*p *>* *.05) different from those of untreated steamed samples (0% PDM, 40.07%) and samples treated with 20% PDM (40.67%) but was significantly (*p *<* *.05) lower than those in samples treated with 40% (41.50%) or 80% PDM (42.70%). For PUFAs, the content in samples treated with 60% PDM increased dramatically from that in samples treated with 40% PDM and showed an additional increase in samples treated with 80% PDM. These unexpected results may be attributed to the instability of fatty acids or to experimental errors. Overall, the changes in fatty acid composition were consistent with the results of lipid oxidation indicators (POV and TBA) in our study, which further demonstrated that PDM may be an effective lipid antioxidant and that addition of PDM may have beneficial effects.

### 3.4 Protein hydrolysis and oxidation

**Figure 2:**
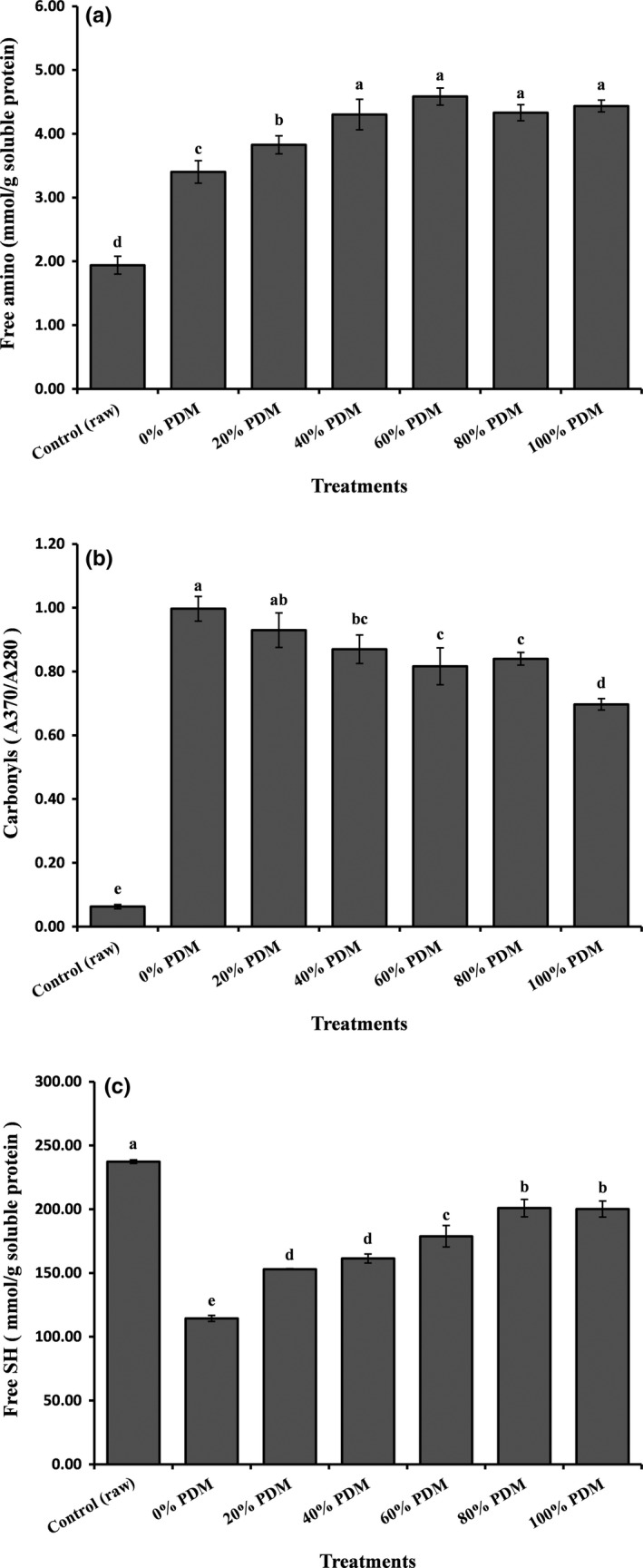
Free amino values (a), carbonyl values (b), and free SH values (c) of steamed pork belly with different levels of added Chinese pickled and dried mustard (PDM). Values are presented as means ± SDs. Bars with different letters are significantly different (*p *<* *.05)

Next, the content of free amino groups was determined to evaluate the degree of protein hydrolysis in pork belly (Figure 2a). The findings showed that the content of free amino groups was 1.94 mmol/g soluble protein in raw pork belly, which was significantly (*p *<* *.05) lower than the values measured for other treatments. This could be explained by the observation that high temperatures promote the heat denaturation of proteins (Zhang, Yao, et al., 2013). Increasing the addition level of PDM from 0% to 40% caused the free amino content to increase from 3.40 to 4.30 mmol/g soluble protein (*p *<* *.05). Additionally, the free amino content remained steady and did not increase significantly (*p *>* *.05) as greater amounts of PDM (40%, 60%, 80%, and 100% PDM) were added. The results showed that PDM promoted the hydrolysis of protein in steamed pork belly and that the degree of protein hydrolysis was dependent on the amount of added PDM within a certain range. This result could be explained by the Maillard reaction in samples, for which glucose from PDM and protein hydrolysates from pork acted as substrates.

The oxidative deterioration of proteins in pork belly treated with PDM was monitored by measuring the amount of carbonyls (Figure 2b) and free sulfhydryl (SH; Figure 2c) groups. Higher carbonyl contents and lower sulfhydryl contents were indicators of a higher degree of protein oxidation (Ali et al., 2015). Notably, for convenience, the carbonyl values in our study were expressed as the ratio of the absorbance at 370 nm to that at 280 nm, equivalent to the carbonyl content per unit protein in the sample. The carbonyl value increased from 0.06 to 1.00, and free SH decreased from 237.29 to 114.43 mmol/g soluble protein after steaming the raw pork belly, indicating that steaming caused significant (*p *<* *.05) protein oxidation in the pork belly sample. This was consistent with the results of Roldan, Antequera, Armenteros, and Ruiz (2014), who showed that the degree of protein oxidation was higher in cooked samples at different time/temperature combinations than in raw samples. After addition of PDM, the degree of protein oxidation was reduced but was significantly (*p *<* *.05) higher than that of raw samples. Moreover, the degree of protein oxidation was negatively correlated with the level of added PDM, to some extent. Among all PDM treatments, the lowest carbonyl value was 0.70 (samples treated with 100% PDM), and the highest value of free SH was 200.90 mmol/g soluble protein (samples treated with 80% PDM). The free SH content in samples treated with 100% PDM (200.18 mmol/g) was not significantly (*p *>* *0.05) different from that in samples treated with 80% PDM. These results clearly showed that addition of PDM affected protein oxidation in steamed pork belly, consistent with the findings of lipid oxidation in the present study. In fact, previous studies have also reported correlations between lipid and protein oxidation. Soyer, Ozalp, Dalmis, and Bilgin (2010) reported that primary and secondary lipid oxidation products could act as substrates for protein oxidation; thus, once the oxidation of lipids started, the oxidation of proteins would also occur. Similarly, the lower degree of protein oxidation in meat could be a result of the lower degree of lipid oxidation inhibited by PDM.

### 3.5 Sensory quality

**Figure 3:**
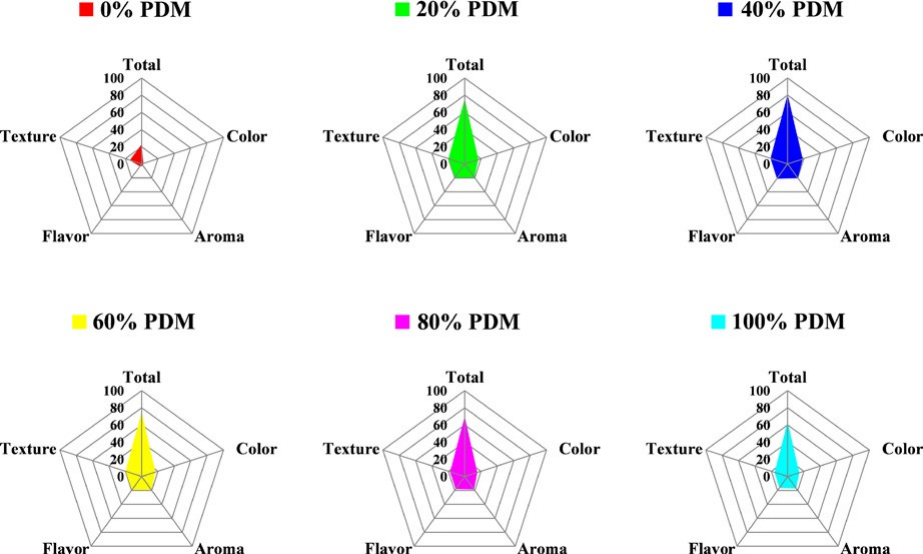
Sensory parameters of steamed pork belly with different levels of added Chinese pickled and dried mustard (PDM). Values are presented as mean scores of each attribute

Sensory qualities (color, aroma, flavor, and texture) of steamed pork belly with different levels of added PDM are shown in Figure 3. The panelists detected significant differences (*p *<* *.01) in the sensory qualities of steamed pork belly samples between samples with and without added PDM, with untreated steamed samples showing the lowest mean score (total = 22.81). Obviously, flavorful PDM is an essential raw material for steamed pork with PDM and contributes to the color, aroma, and flavor of the dish. The total mean score increased from 75.69 to 80.56 when the level of added PDM increased from 20% to 40% and decreased thereafter to 63.50 when 100% PDM was added. Moreover, the four sensory quality attributes showed the same trend as the total mean score. Thus, the sensory quality of steamed pork belly was the best when 40% PDM was added; in these samples, the highest mean scores were observed for each attribute (color = 18.50, aroma = 20.06, flavor = 21.00, and texture = 21.00). When the amount of PDM was lower, steamed pork belly did not exhibit a sufficient brown color, typical potherb mustard pickled flavor, or acidic taste (Zhao, Tang, & Ding, 2007). Additionally, the relatively high amount of meat fat (not enough absorbed by PDM) could make people feel sick. In contrast, when too much PDM was added, the aroma and flavor of the steamed pork belly were not as apparent, and the dish appeared too dark in color. Moreover, loss of moisture and fat caused the texture to be too tough, similar to the results of Lorido, Ventanas, Akcan, and Estevez (2016), who showed that the texture of products was often related to their composition (moisture and intramuscular fat content). Additionally, the results of texture profile analysis showed that both muscle and fat layers of steamed pork belly had the lowest hardness, gumminess, and chewiness values in samples treated with 40% PDM compared with those in other groups; thus, the meat tasted soft, smooth, and tender in these samples (data not shown). In summary, moderate addition of PDM (40%) yielded the best sensory qualities.

### 3.6 Shelf life

The OXITEST reactor, a new instrument used to evaluate the shelf life of samples rich in lipids by monitoring oxygen consumption, was used to determine the oxidative stability of pork belly samples. Using this technique, food stability against rancidity can be measured directly in whole foods (solid, liquid, and dough) without the requirement for fat separation, and the information obtained from the instrument pertains not only to the IP of the autoxidation process but also to the rate and acceleration of the autoxidation process and to the amount of oxygen consumed by the product (under specific accelerated conditions) (Verardo et al., 2013).

Pork belly samples with different treatments were determined under three induced temperatures (80, 90, and 100°C), and the IPs are shown in Table 3. Our findings showed that the natural logarithm of IP was linearly related to the induced temperature (*R* ^2 ^> .99). The slope was negative, and the intercept was positive for all straight lines, suggesting that the IP was reduced, while the oxidative stability was decreased as the temperature increased such that the samples would be preserved for shorter times. This was in accordance with reports by Saeed and Howell (2002) and Soyer et al. (2010), demonstrating that low storage temperature reduced the lipid and protein oxidation of meat during frozen storage. Based on the linear model calculated by the OXITEST reactor, IPs under room temperature at 25°C (IP25) were estimated to more intuitively evaluate the shelf life of samples at the same induced temperature. Steaming pork belly without adding PDM (untreated steamed samples, IP25 = 20:11:16, days:hr:min) was estimated to have a shorter IP than raw samples at room temperature (IP25 = 36:07:46, days:hr:min). These findings may be explained by the induction of lipid and protein oxidation during the heating process in cooked samples (Roldan et al., 2014), resulting in weaker oxidative stability than that of raw meat. Moreover, PDM treatment affected the IP values of pork belly samples by changing the gradients of lines in the model. As the amount of added PDM increased (from 20% to 100%), the IP25 values also increased (from 78:09:56 to 133:18:54, days:hr:min), indicating longer sample preservation times. The results showed that the oxidative stability of steamed pork belly was positively influenced by PDM, corresponding to the results of lipid and protein oxidation in our study.

**Table 3:** Effects of Chinese pickled and dried mustard on induction periods (IPs) of steamed pork belly

Treatments	IPs (hr)—T (°C)	*R* ^2^	Estimated IP25 (days:hr:min)
Control (raw)	ln(IP) = −0.091343T + 9.054094	.9996	36:07:46
0% PDM	ln(IP) = −0.084285T + 8.304128	1.0000	20:11:16
20% PDM	ln(IP) = −0.091973T + 9.839377	.9956	78:09:56
40% PDM	ln(IP) = −0.092739T + 9.960327	.9966	86:19:37
60% PDM	ln(IP) = −0.093378T + 10.082305	.9999	96:12:37
80% PDM	ln(IP) = −0.094660T + 10.235775	.9995	108:23:38
100% PDM	ln(IP) = −0.094824T + 10.444899	.9987	133:18:54

PDM, pickled and dried mustard.

## 4 CONCLUSIONS

In the Chinese traditional dish, steamed pork with PDM, PDM plays an essential role in nutritional quality, sensory quality, and shelf life of steamed pork belly. In this study, we found that PDM decreased the moisture and total lipid contents, but increased the crude protein, ash, and sodium chloride contents in steamed pork belly. In addition, lower TBA and POV values as well as higher UFA/SFA ratios in PDM‐treated samples were observed, confirming that PDM effectively reduced lipid oxidation in steamed pork belly. PDM also promoted protein hydrolysis and inhibited protein oxidation of steamed pork belly to some degree. Therefore, the OXITEST analysis showed that steamed pork belly had a longer shelf life with higher addition of PDM under the same induced temperature. Furthermore, all of the changes in chemical composition in response to addition of PDM could affect the sensory and nutritional qualities of steamed pork belly. Although 100% PDM treatment could maximize the shelf life of the samples, the optimal sensory qualities of steamed pork belly were achieved by the addition of 40% PDM according to TPA and sensory evaluation. These results provided a theoretical basis for the preservation mechanism of PDM. However, further studies are required for evaluating the optimal sensory characteristics of the dish prepared with 40% PDM.

## CONFLICT OF INTEREST

The authors declare that they do not have any conflict of interest.

## ETHICAL REVIEW

This study does not involve any human or animal testing.

## INFORMED CONSENT

Written informed consent was obtained from all study participants.
